# Characterization of adhesion between *Limosilactobacillus reuteri* and milk phospholipids by density gradient and gene expression

**DOI:** 10.3168/jdsc.2020-18939

**Published:** 2020-10-29

**Authors:** Lin Zhang, Israel García-Cano, Rafael Jiménez-Flores

**Affiliations:** Department of Food Science and Technology, Parker Food Science & Technology Building, The Ohio State University, Columbus 43210

## Abstract

The benefits of fermented dairy products, in particular the presence of lactic acid bacteria (LAB) and milk phospholipids (MPL), seem to correlate with positive effects on human health. We hypothesize that one aspect of this benefit is the adhesion of LAB to the milk fat globule membrane via the interaction of LAB and MPL. Our first objective was to present a method to characterize and quantify such adhesion and investigate its association with a physical test. Our second objective was to further analyze the mechanism of interaction by analyzing expression of 3 previously reported surface binding-promoting genes (*MapA, Cnb*, and *CmbA*). We categorized adhesion between MPL and LAB by observing the distribution of MPL in corresponding bacterial cultures. Our working hypothesis was that any interaction or adhesion between these 2 components would yield differences in the distribution of MPL. Out of 122 LAB tested, 27% showed what could be characterized as adhesion; 38% of these strains were *Limosilactobacillus reuteri*. Further characterization of adhesion was carried out using an reverse transcription quantitative-PCR experiment, which demonstrated that the relative expression level of *CmbA* was positively associated with that adhesion. In addition, supplementation of MPL caused overexpression of *MapA* and *Cnb* in *L. reuteri* OSU-PECh-37A and OSU-PECh-48. This study indicated strain-specific adhesion between MPL and LAB and suggested that *CmbA*, which encodes a surface protein, is a potential factor involved in that adhesion. A better understanding of interactions between MPL and LAB may contribute to the design of new functional products and improve the delivery of these bioactive ingredients to their target site of action.

## Highlights

• The interaction between lactic acid bacteria and milk phospholipids can be semi-quantified
• Binding and interaction between milk phospholipids and LAB is mediated by gene modulation
• Two of three genes for surface adhesion corresponded directly with binding results
• This method identifies LAB that adhere tightly to the intestinal membranes

## Graphical Abstract

**Figure:**
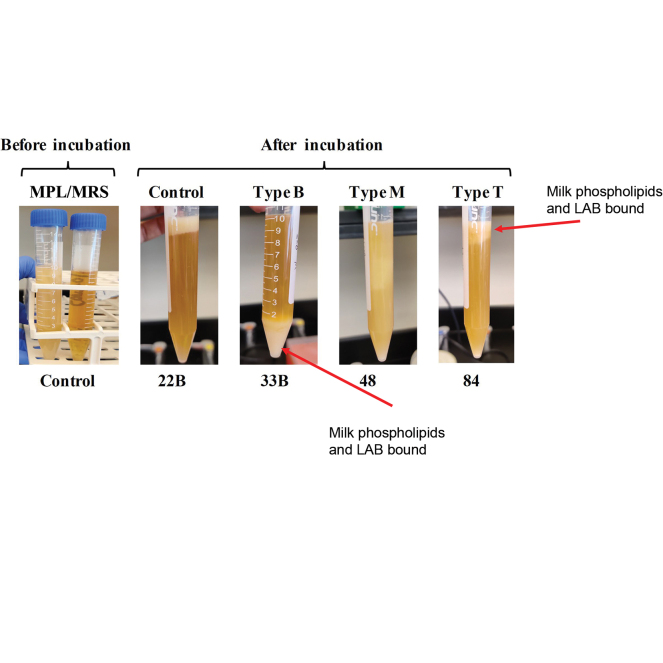
Summary: Adhesion between lactic acid bacteria (LAB) and milk phospholipids may be correlated with positive effects on human health. We present a method to characterize and quantify potential adhesion between lactic acid bacteria and milk phospholipids with a physical test. The association between such adhesion and the expression of surface binding-promoting genes of *Limosilactobacillus reuteri* has been analyzed. Our results lead to a better understanding of the interaction between lactic acid bacteria and milk phospholipids and contribute to the development of fermented dairy products supplemented with milk phospholipids.

Lactic acid bacteria (**LAB**) have been associated with many beneficial effects on human health. Dairy products are traditionally considered ideal vehicles for the delivery of LAB and maintain optimal conditions for their health benefits (Gomand et al., 2019). Interactions between LAB and dairy components such as protein and fat affect their distribution in food such as fermented milk and cheese, promote the growth of LAB, and help retain bacterial viability against gastric conditions (Burgain et al., 2014; Singh et al., 2016; Gomand et al., 2018). Some LAB have been observed to preferentially associate with the fat or protein interface in cheese products, which contributes to the quality of cheese (Lopez et al., 2006). Direct adhesion between *Limosilactobacillus reuteri* and milk fat globule membrane (**MFGM**) has also been observed, and the level of adhesion has been determined to vary with conditions and strain of bacteria (Brisson et al., 2010). Milk phospholipids (**MPL**), major components of the MFGM, have been associated with many health benefits as well, including promoting cognitive development (Ortega-Anaya and Jiménez-Flores, 2019) and intestinal development, protecting against “leaky gut” syndrome (Snow et al., 2011) and inflammation (Bhinder et al., 2017), and inhibiting cancer cell proliferation (Castro-Gómez et al., 2016; Huërou-Luron et al., 2018). The major MPL in bovine milk are phosphatidylethanolamine, phosphatidylcholine, sphingomyelin, phosphatidylserine, and phosphatidylinositol (Ortega-Anaya and Jiménez-Flores, 2019). A recent study found that MPL-treated LAB showed higher adhesion to Caco-2 cells, which might be affected by the adhesion between LAB and MPL (Rocha-Mendoza et al., 2020). Increased adherence gives those LAB better adherence competence against other bacteria.

Because of the health benefits of MPL and LAB, the application of supplementing MPL into LAB-fermented dairy products is garnering the attention of scientists and has shown some synergistic effects. A previous study found that co-ingestion of sphingomyelin and LAB-fermented milk significantly enhanced the absorption and bioavailability of dietary sphingomyelin in rats (by 2-fold) compared with consuming fermented milk alone (Amagai et al., 2017). Fermented milk supplemented with MPL has been shown to improve the enteric environment and skin conditions of dogs with allergic skin disorders (Morifuji et al., 2017). The potential binding between LAB and MPL has not been fully studied or characterized. A better understanding of LAB–MPL adhesion may contribute to the design of new functional products and improve the delivery of both bioactive ingredients to their target site of action.

During our preliminary experiment, we observed differences in the distribution of supplemented MPL in bacterial cultures, which might be caused by LAB–MPL adhesion. According to Brisson et al. (2010), adhesion might be affected by the presence of surface proteins that mediate the binding of LAB to some extracellular matrices. Surface proteins of *L. reuteri*, including the mucus adhesion-promoting protein (**MapA**), the collagen-binding protein (**Cnb**), and a putative sortase-dependent cell and mucus binding protein (**CmbA**), have been shown to promote the adhesion of bacteria to environmental components (Miyoshi et al., 2006; Hsueh et al., 2010; Jensen et al., 2014). The objectives of this study were to categorize and quantify potential adhesion between MPL and LAB and to investigate the association of adhesion with expression of 3 surface binding-promoting genes of *L. reuteri*: *MapA, Cnb*, and *CmbA*. We hypothesized that different LAB adhere, with some specificity, to MPL, that this adhesion may vary in “strength of binding,” and that it may be strain-dependent (i.e., associated with differential gene expression of different surface proteins). Here, we present results for some chosen proteins selected from the literature that can demonstrate our hypothesis.

The LAB strains tested for adhesion were previously isolated from commercial fermented dairy products and deposited in the OSU-PECh culture collection (The Ohio State University, Columbus; García-Cano et al., 2019). Fresh bacterial culture was normalized to an optical density of 2.0 at 600 nm (**OD_600_**) using a spectrophotometer reader (Multiskan GO, Thermo Scientific, Waltham, MA) and then inoculated 1:100 (vol/vol) into 10 mL of de Man, Rogosa, and Sharpe medium (**MRS**; Difco/Becton Dickinson and Co., Sparks, MD) as the control (untreated) or MRS medium supplemented with 1% (wt/vol) MPL (Fonterra, Auckland, New Zealand; MRS + 1% MPL) as the treatment. The cultures were incubated at 37°C for 24 h without shaking. The binding between MPL and 122 strains of LAB were observed experimentally. Those 122 strains included 36 *Lacticaseibacillus rhamnosus*, 24 *L. reuteri*, 10 *Lactobacillus helveticus*, 10 *Lactobacillus gasseri,* 8 *Lacticaseibacillus paracasei*, 6 *Lactobacillus acidophilus*, 6 *Lacticaseibacillus casei*, 5 *Lactobacillus amylolyticus*, 3 *Lactobacillus johnsonii*, 3 *Lactobacillus crispatus*, 2 *Lactobacillus delbrueckii*, 2 *Pediococcus pentosaceus*, 2 *Pediococcus acidilactici*, 1 *Lactobacillus amylovorus*, 1 *Lactiplantibacillus pentosus, 1 Enterococcus faecium*, 1 *Enterococcus mundtii*, and 1 *Streptococcus thermophilus*. The bacteria were classified into 3 categories based on the distribution of the MPL layer in corresponding bacterial cultures after the incubation: type B, which caused MPL to settle to the bottom of the tube; type M, which caused MPL to distribute throughout the tube; and type T, which caused MPL to float to the top of the tube (Figure 1A).

**Figure 1:**
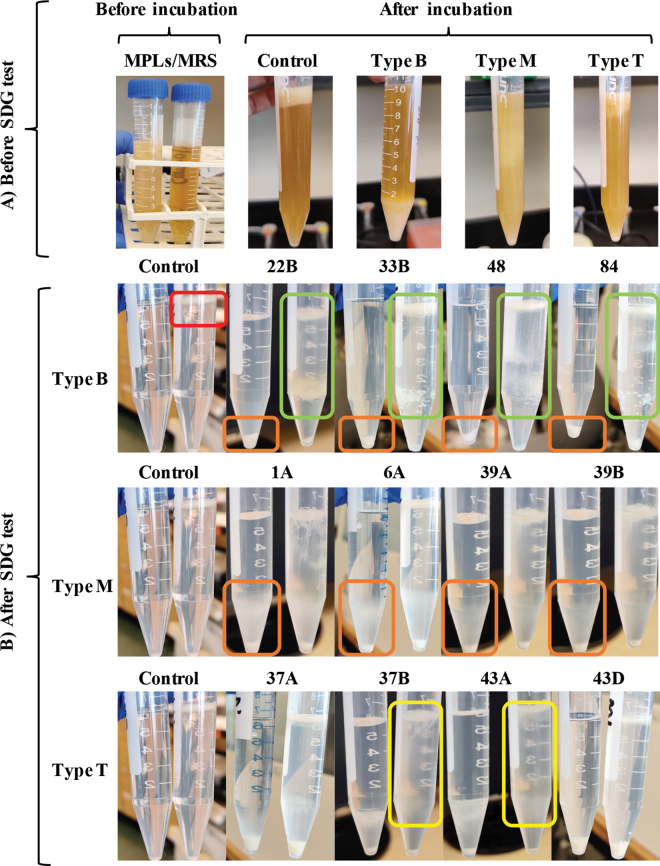
(A) Observation of bacterial cultures before and after incubation in de Man, Rogosa, and Sharpe (MRS) medium with or without milk phospholipids (MPL). Type B bacteria caused MPL to settle at the bottom, type M bacteria caused MPL to distribute throughout the tube, and type T bacteria caused MPL to float to the top. (B) Tubes after removal of MRS broth and following sucrose density gradient (SDG) centrifugation test with pellets from tubes previously containing MRS on the left, and those previously containing MRS supplemented with 1% MPL on the right. Supplemented MPL floated to the top of the solution (red square), most bacteria pellets cultured in MRS settled at the bottom of tubes (orange squares), and bacterial pellets moved to the upper sucrose solution layer (green and yellow squares). Strains: 22B = *Lactobacillus crispatus* (OSU-PECh-22B); 33B = *Limosilactobacillus reuteri* (OSU-PECh-33B); 48 = *L. reuteri* (OSU-PECh-48); 84 = *L. reuteri* (OSU-PECh-84); 1A = *Pediococcus acidilactici*(OSU-PECh-1A); 6A = *Pediococcus lolii* (OSU-PECh-6A); 39A = *L. reuteri* (OSU-PECh-39A); 39B = *Enterococcus faecium*(OSU-PECh-39B); 37A = *L. reuteri* (OSU-PECh-37A); 37B = *Lacticaseibacillus rhamnosus* (OSU-PECh-37B); 43A = *L. reuteri* (OSU-PECh-43A); 43D = *Lacticaseibacillus rhamnosus* (OSU-PECh-43D).

After incubation, we used a sucrose density gradient (**SDG**) centrifugation test modified from Brisson et al. (2010) to quantify adhesion of 12 LAB strains, containing 6 randomly selected *L. reuteri* (OSU-PECh-33B, OSU-PECh-37A, OSU-PECh-39A, OSU-PECh-43A, OSU-PECh-48, and OSU-PECh-84) and 6 strains from other species: *Lactobacillus crispatus* (OSU-PECh-22B); *Lacticaseibacillus rhamnosus* (OSU-PECh-37B, OSU-PECh-43D); *Pediococcus acidilactici*(OSU-PECh-1A); *Pediococcus lolii* (OSU-PECh-6A); and *Enterococcus faecium*(OSU-PECh-39B), so that we had 4 strains from each defined category (types B, M, and T). For both control and treatment samples, 10 mL of fully grown bacterial culture was homogenized by vortexing and then divided into two 5-mL portions. The portions sent for the SDG centrifugation were labeled A for the control and B for the treated sample; the portions used for estimating initial bacterial concentration without SDG centrifugation were labeled A′ for the control and B′ for the treated sample. All tubes were first centrifuged at 3,578 × *g* at 4°C for 30 min (TX1000 Swinging Bucket Rotor; Sorvall Legend XF, Thermo Scientific). After the first centrifugation, the MRS broth supernatant was decanted, which left a bacterial pellet in control tubes A and A′ but a mixture pellet containing both MPL and grown bacteria in treatment tubes B and B′. The pellets from tubes A′ and B′ were washed 1 time (3,578 × *g* for 10 min) with 20% sucrose solution and 3 times (3,578 × *g* for 10 min) with PBS (pH 7.4) to remove the remaining MRS broth and then resuspended in 1 mL of PBS. The absorbance of the suspension at 600 nm (Fisherbrand AccuSkan GO UV/Vis Microplate Spectrophotometer, Fisher Scientific, Pittsburgh, PA) was measured to represent the initial bacterial concentration [**OD_600(A_**_′_**_)_** and **OD_600(B_**_′_**_)_**, respectively]. In tubes A and B, 2 mL of each concentration of sucrose solution (60 > 40 > 20%) was added slowly, followed immediately by centrifugation (3,578 × *g* at 4°C for 30 min).

After centrifugation, the supernatant of all tubes was decanted. The remaining bacterial pellet in each tube was resuspended in 1 mL of PBS, and the bacterial concentration was measured [**OD_600(A)_** and **OD_600(B)_**, respectively]. The loss in bacteria in the control sample caused by treatment-irrelevant factors such as loss during handling and the nature of bacteria was calculated as follows:

$$1-\frac{{\text{OD}}_{600\left(\text{A}\right)}}{{\text{OD}}_{600\left({\text{A}}^{\prime }\right)}},$$

whereas the loss in bacteria in the treated sample caused by binding to MPL and other treatment-irrelevant factors was calculated as follows:

$$1-\frac{{\text{OD}}_{600\left(\text{B}\right)}}{{\text{OD}}_{600\left({\text{B}}^{\prime }\right)}}.$$.

The loss in bacteria solely due to the specific interaction between bacteria and MPL was thus calculated as

$$\left(1-\frac{{\text{OD}}_{600\left(\text{B}\right)}}{{\text{OD}}_{600\left({\text{B}}^{\prime }\right)}}\right)-\left(1-\frac{{\text{OD}}_{600\left(\text{A}\right)}}{{\text{OD}}_{600\left({\text{A}}^{\prime }\right)}}\right).$$.

Tubes with MRS and MRS + 1% MPL were used as blanks. Triplicates were performed for all tested bacteria. The extent of bacteria adhered to MPL (adhesion %) was calculated using the following equation:

$$\text{Adhesion}=\left(\frac{{\text{OD}}_{600\left(\text{A}\right)}}{{\text{OD}}_{600\left({\text{A}}^{\prime }\right)}}-\frac{{\text{OD}}_{600\left(\text{B}\right)}}{{\text{OD}}_{600\left({\text{B}}^{\prime }\right)}}\right)\times 100\%,$$

where OD_600(A)_ and OD_600(B)_ refer to the absorbance at 600 nm of the remaining bacterial suspensions after the SDG test of bacteria previously cultured in MRS (control) and MRS + 1% MPL (treatment) media, and OD_600(A′)_ and OD_600(B′)_ refer to the absorbance at 600 nm of the original bacterial suspensions (no SDG test).

Six *L. reuteri* strains were analyzed using reverse transcription quantitative-PCR. Of those 6 strains, 3 were randomly selected from type B *L. reuteri* (OSU-PECh-33B, OSU-PECh-48, and OSU-PECh-84), and the other 3 were randomly selected from types other than type B: *L. reuteri* OSU-PECh-37A, OSU-PECh-39A, and OSU-PECh-43A. The RNeasy Plus Mini Kit (Qiagen, Valencia, CA) and the iScript Reverse Transcription Supermix Kit (Bio-Rad, Hercules, CA) were used as instructed by the respective manufacturers to conduct total RNA extraction and cDNA synthesis, respectively. The RT-qPCR reaction mix was prepared using the iQ SYBR Green Supermix (Bio-Rad) with 50 ng of cDNA template. The RT-qPCR was performed in the C1000 Touch Thermal Cycler (Bio-Rad) and analyzed using the ΔCq method (Bio-Rad). The primer sequences for *MapA* were forward 5′-CCGGTCTTGGTTCAGGTAAG-3′, reverse 5′-ATGCAAACCGGGACTTGATA-3′; for *Cnb*, forward 5′-TTGCTGGGACAGGAACTAATAAT-3′, reverse 5′-CCGACCTTGCTTGATCATATCT-3′; for *CmbA*, forward 5′-CCAACGGCAGTAGGAACTTATC-3′, reverse 5′-GGTGTCTGTGCTGGCTTAAT-3′; with universal primer as the reference gene, forward 5′-AGCAGTAGGGAATCTTCCA-3′, reverse 5′-CACCGCTACACATGGAG-3′ (Walter et al., 2000; Heilig et al., 2002). The quantitative PCR conditions were as follows: 1 cycle at 95°C for 3 min and 40 cycles at 95°C for 10 s and 55°C for 1 min. Three biological replicates and 3 experimental replicates were performed.

All statistical analyses were performed using SAS software (version 9.4, SAS Institute Inc., Cary, NC). Adhesion quantification data were analyzed using one-way ANOVA, followed by Tukey's test. The relative gene fold change induced by MPL supplementation was analyzed using the Wilcoxon signed-rank test, with a 2-fold change considered significant. The Kendall rank correlation coefficient was used to evaluate the association between intrinsic relative gene expression level and adhesion. The criterion for the significance of all tests was set at *P* < 0.05. Data are reported as means ± standard deviations.

For the 122 bacterial cultures, MPL and bacterial pellets settled in 27% of LAB cultures (type B), were suspended throughout the tube in 28% (type M, indicating the bacteria might bind to MPL weakly or randomly), and floated to the top in the rest (45%; type T, indicating no interaction between bacteria and MPL) (Figure 1A). *Limosilactobacillus reuteri* (38%) were the major species in which MPL settled to the bottom of the bacterial cultures and the second major species in the OSU-PECh culture collection. Fifteen out of 24 *L. reuteri* tested showed that property. Adhesion between the MPL-containing ingredient (MFGM) and *L. reuteri* has been studied previously (Brisson et al., 2010); the interaction was observed to be strain-specific and bacterial surface hydrophobicity was found to play a role in the interactions between MFGM and *L. reuteri* (Brisson et al., 2010; Guerin et al., 2019). Cell hydrophobicity depends on various components, including surface proteins, exopolysaccharides, peptidoglycans, and lipoteichoic acids (Guerin et al., 2019). Brisson et al. (2010) found that a higher cell surface hydrophobicity in some strains of *L. reuteri* was associated with a higher level of LiCl-extractable cell-surface proteins. Therefore, in our study, *L. reuteri* with unique surface properties (e.g., the presence of some cell-surface proteins) might adhere to MPL and pull MPL to the bottom of the tube because of the increased density of the LAB–MPL complex. This finding indicates the different extents or types of adhesion or interactions between MPL and LAB. These interactions induced differences in distribution of MPL in corresponding bacterial cultures. We focused on the final location of the MPL because it was easier to observe than bacteria cells.

The SDG test, which separates particles by centrifugal force but maintain them at a given place in the tube based on their density difference, allowed for separation of any unbound bacteria, which had higher density than the MPL; bacteria bound to MPL had intermediate density. After the SGD test, in tubes previously containing pure medium, the sucrose solution was clear, and the MPL layer (if any) floated to the top (Figure 1B control, red square) because of its lower density (lower than the sucrose solution). We observed that most type B bacteria migrated to the upper sucrose layer (Figure 1B, green square), some type M bacteria migrated to the upper layer (Figure 1B), and a few type T bacteria moved to the upper sucrose layer (Figure 1B, yellow square). Bacterial strains previously cultivated in MRS were run as a control to confirm that most bacteria without MPL treatment were dense enough to form a pellet at the bottom of the tube (Figure 1B, orange square). This process was done in every experiment to make comparisons easier. We also observed that some untreated bacteria could migrate up to the 60% sucrose layer (Figure 1B, orange square) because of uncontrolled factors other than their interaction with MPL, which could lead to bacterial loss after decanting. Thus, we took that into consideration, and the loss in bacterial concentration due to other factors was subtracted out in the calculation. The binding of bacteria to MPL, calculated as adhesion percentage, is presented in bar graph form (Figure 2). The adhesion percentage between MPL and type B LAB differed from that between MPL and type M and T bacteria (*P* < 0.001), whereas the latter 2 did not differ significantly from each other (*P* > 0.05; Figure 2). The SDG test confirmed that the MPL that settled to the bottom with the bacteria indicated stronger adhesion between MPL and type B bacteria. Also, adhesion was shown to be strain-specific: we demonstrated a distinction between a type B strain (*L. reuteri* OSU-PECh-48, identified as *L. reuteri* strain MG505) and a type T strain (*L. reuteri* OSU-PECh-37A, identified as *L. reuteri* strain DSM 108836) of the same species, as characterized by 16S rRNA sequencing.

**Figure 2:**
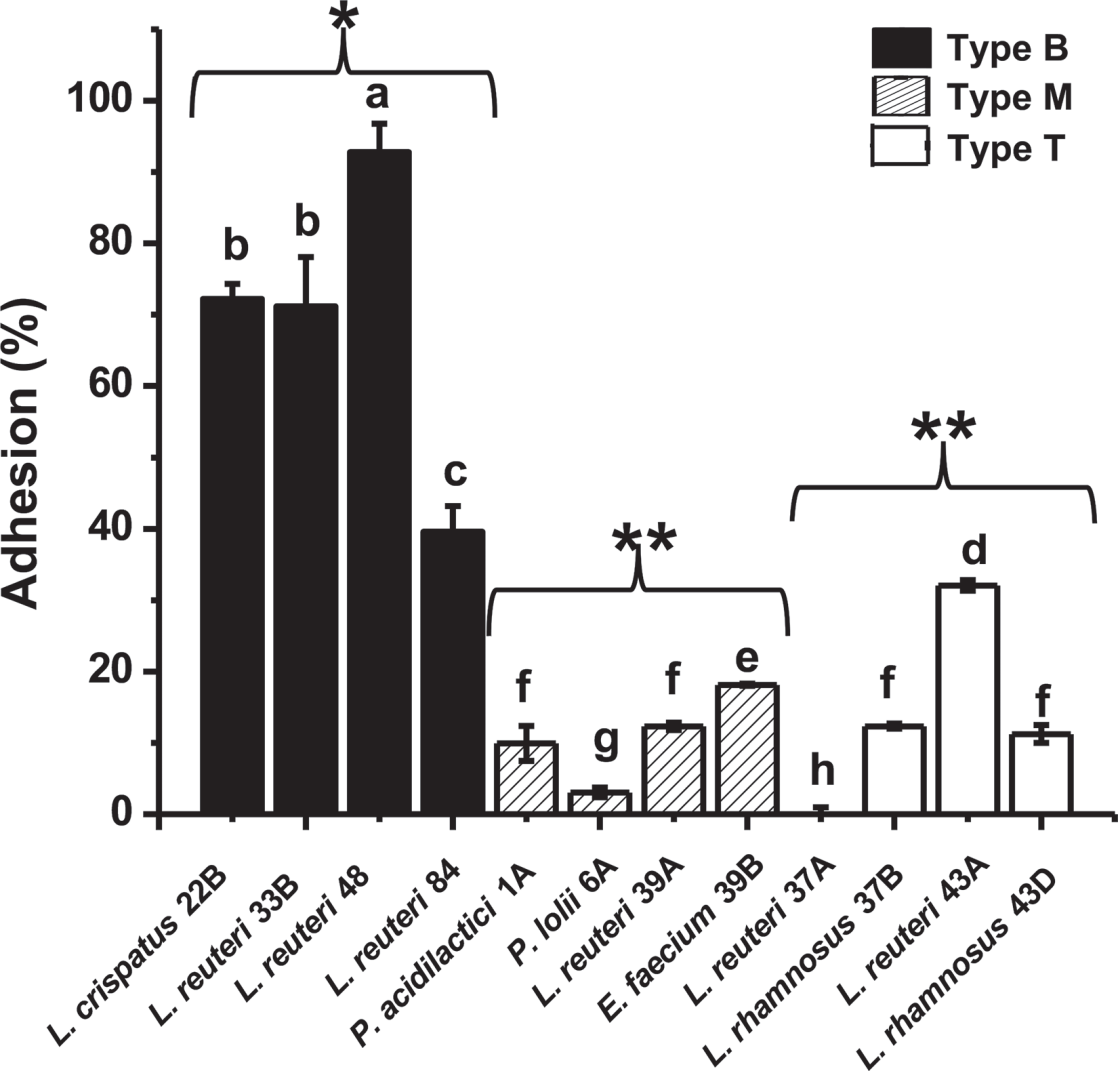
Adhesion (%) of 12 lactic acid bacteria (LAB) species to milk phospholipids (MPL). Lowercase letters (a–h) indicate significant differences (*P* < 0.05) between adhesion percentage and MPL in different LAB. * indicates significant differences (*P* < 0.05) between adhesion percentage and MPL in each type of LAB; **indicates a nonsignificant difference between type T and M. Type B bacteria caused MPL to settle at the bottom, Type M bacteria caused MPL to distribute throughout the tube, and Type T bacteria caused MPL to float to the top. Strains: *Lactobacillus crispatus* 22B; *Limosilactobacillus reuteri* 33B; *L. reuteri* 48; *L. reuteri* 84; *Pediococcus acidilactici* 1A; *Pediococcus lolii* 6A; *L. reuteri* 39A; *Enterococcus faecium* 39B; *L. reuteri* 37A; *Lacticaseibacillus rhamnosus* 37B; *L. reuteri* 43A; *Lacticaseibacillus rhamnosus* 43D. Error bars represent standard deviations.

Adhesion between LAB and MPL is affected by the balance between the appearance of exopolysaccharides and surface roughness, surface free energy and hydrophobicity of bacteria and MPL, intermolecular forces (e.g., electrostatic interactions, van der Waals, and Lewis acid/base interactions), and environmental factors (temperature and pH; Burgain et al., 2014). The many types of interaction between LAB and MPL make it difficult to determine the factors involved in adhesion because MPL supplementation would affect the cell wall composition of LAB (Burgain et al., 2014). The close contact of LAB and MPL might enhance the metabolism of MPL, thereby affecting the surface properties of supplemented MPL (Ly et al., 2006). In addition, metabolites produced by fermentation, including acid and exopolysaccharides, would change the pH and viscosity of the culture medium, which in turn affect adhesion between LAB and MPL (Burgain et al., 2014). In the present study, we controlled the culture medium, bacterial inoculum, and fermentation conditions (temperature and time); therefore, all observed differences in adhesion between LAB and MPL were induced by specific interactions between MPL and the individual LAB strain, such as MPL-induced gene expression change, and intrinsic characteristics of the LAB strain, such as the presence of binding-promoting proteins.

In addition to the SDG test, Gomand et al. (2018) developed a new approach using bacterial immobilization for high-throughput screening of adhesion between *L. rhamnosus* GG and dairy biomolecules. Adhesion between MPL and LAB can be further visualized at the microscopic level using confocal laser scanning microscopy, scanning electron microscopy, or atomic force microscopy, which have been used to observe the location of LAB in various cheeses and the interactions between *L. rhamnosus* GG and isolated whey proteins (Lopez et al., 2006; Brisson et al., 2010; Guerin et al., 2016). Other methods, such as optical tweezers used to measure the binding force between bacteria and other components, can be coupled with the SDG test to quantify the adhesion between LAB and MPL (Brisson et al., 2010).

The RT-qPCR experiment allowed us to test whether the relative gene expression level of selected genes in different bacteria was associated with adhesion. The Kendall rank correlation coefficients (τ) indicated that *MapA* and *Cnb* were unlikely to be associated with adhesion in *L. reuteri* (τ*_MapA_* = −0.2418, *P_MapA_* = 0.1751; τ*_Cnb_* = −0.118, *P_Cnb_* = 0.4951). However, for *CmbA*, τ_CmbA_ = 0.7997 and *P*_CmbA_ < 0.01 demonstrated that the relative expression of *CmbA* was positively associated with binding to MPL in the *L. reuteri* strains tested. The *CmbA* gene encodes cell and mucus binding protein A (CmbA) and belongs to a group of surface-associated proteins in gram-positive bacteria (Jensen et al., 2014). The CmbA protein contains an N-terminal signal peptide with a YSIRK-G/C motif that can direct the protein to a specific surface localization, a C-terminal LPxtG motif followed by a hydrophobic region predicted to be a transmembrane helix, and a positively charged tail (Jensen et al., 2014). Protein CmbA from *L. reuteri* ATCC PTA 6475 has been found to promote adhesion between the bacteria and mucus and intestinal epithelial cells, and it is unique to some *L. reuteri* strains (Jensen et al., 2014). In our study, as confirmed by cycle quantification (Cq) values, CmbA is either absent or expressed at a low level in non-type-B bacteria *L. reuteri* OSU-PECh-37A, OSU-PECh-39A, and OSU-PECh-43A, which showed low adhesion with MPL compared with the type B *L. reuteri* strains tested (OSU-PECh-33B, OSU-PECh-48, and OSU-PECh-84; Figure 3). Although CmbA is absent or expressed at a low level in *Lactobacillus crispatus* 22B, this type B bacterium also showed high adhesion with MPL, indicating that other unknown proteins or factors could play a role in mediating binding with MPL in other non-*L. reuteri* bacteria such as *Lb. crispatus*.

**Figure 3:**
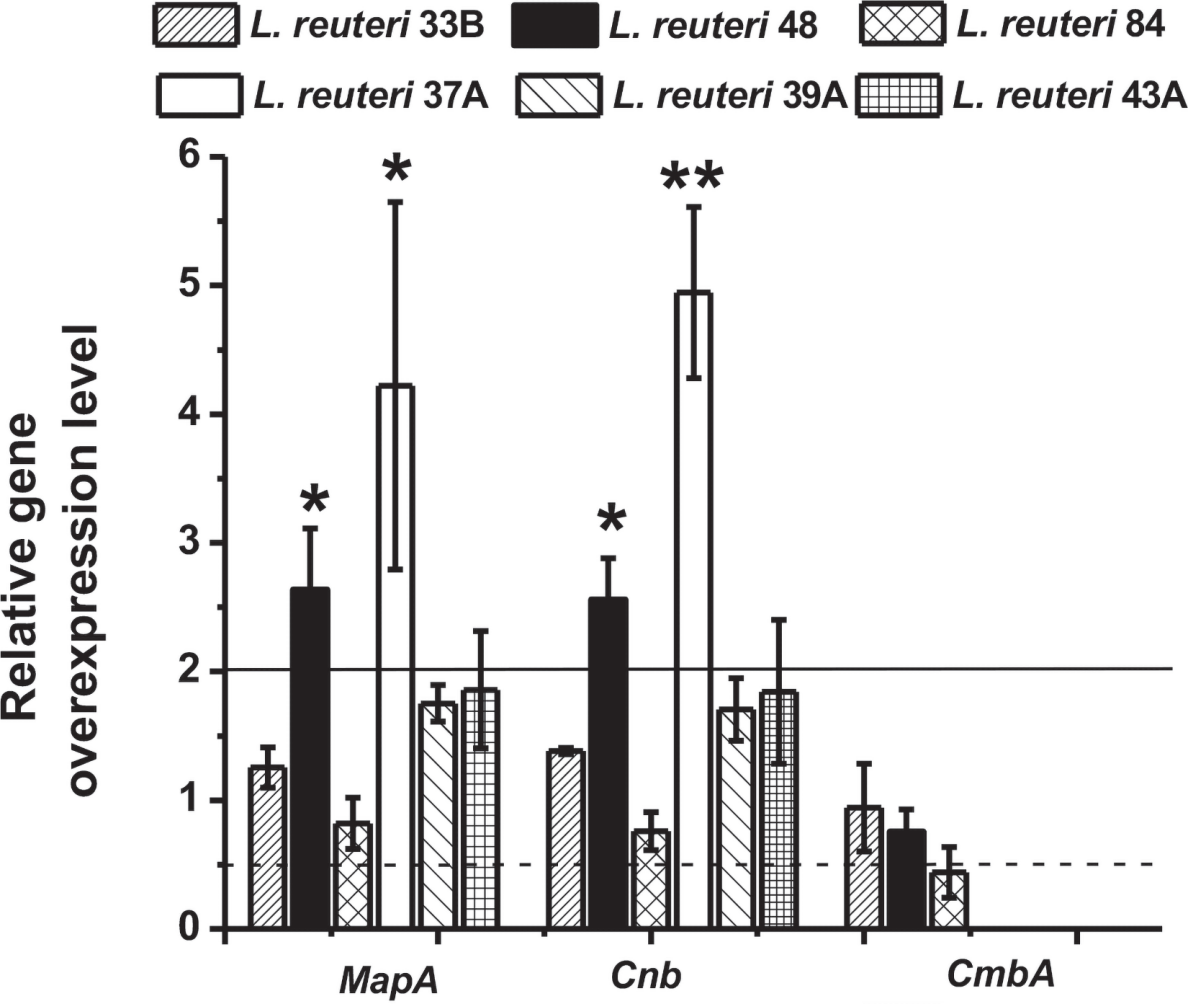
Relative overexpression level of genes *MapA, Cnb*, and *CmbA* in 6 strains (33B, 48, 84, 37A, 39A, and 43A) of *Limosilactobacillus reuteri* with milk phospholipids (MPL) supplementation. * indicates a mean value significantly greater than 2.0 (*P* < 0.05), which indicates the gene has been overexpressed more than 2-fold; ** indicates that the level of expression of *Cnb* was significantly different (*P* < 0.05) from that of *MapA*. Error bars represent standard deviations.

The Wilcoxon signed-rank test indicated that addition of 1% MPL into MRS medium did not significantly upregulate or downregulate the expression of the tested genes (when a 2-fold change was considered significant) in *L. reuteri* in general (*P* > 0.05). The relative gene expression levels of *MapA* and *Cnb* showed similar trends, consistent with the previous finding that *MapA* and *Cnb* are considered homologs because of their high similarity at the amino acid level (94%; Miyoshi et al., 2006). However, *MapA* and *Cnb* of *L. reuteri* OSU-PECh-37A and OSU-PECh-48 were overexpressed with supplementation of 1% MPL. Proteins encoded by *MapA* and *Cnb* are known to be adhesion factors in *L. reuteri* 104R, promoting the adherence of the bacteria to the intestinal mucosa (Miyoshi et al., 2006). Further, MapA could compete with other adhesion proteins (e.g., periplasmic amino acid-binding protein, Peb1) of *Campylobacter jejuni*, which would contribute to preventing or remedying gastrointestinal infectious disease in humans (Miyoshi et al., 2006). A previous study found that LAB pretreated with MPL had higher attachment to Caco-2 cells (Rocha-Mendoza et al., 2020). The overexpression of *MapA* and *Cnb* induced by MPL supplementation suggests that MPL-supplemented fermented dairy products may mediate the adherence of some LAB to Caco-2 cells and mucus because adherence is controlled by adhesion factors such as MapA and Cnb, which may increase their competency against other bacteria and help regulate gut health.

The distribution of supplemented MPL in bacterial cultures beyond the exponential phase was categorized by observation: settled at the bottom, suspended throughout the tube, or floating at the top of the tube. Medium supplemented with MPL that settled to the bottom indicated strong strain-specific adhesion between MPL and corresponding LAB. The SDG test enabled us to quantify adhesion between LAB and MPL by separating unbound bacteria (higher density) from MPL (lower density) and LAB bound to MPL (intermediate density). As confirmed by the SDG test and gene expression analysis, adhesion between *L. reuteri* and MPL was associated with expression of *CmbA*. In addition, supplementation of MPL into MRS was associated with overexpression of *MapA* and *Cnb* in *L. reuteri* OSU-PECh-37A and OSU-PECh-48, indicating that MPL supplementation might extend the residence time of those strains in the gut and improve their competitiveness by promoting their adherence to the intestinal mucosa. The study leads to a better understanding of interactions between LAB and MPL and contributes to the development of novel MPL-supplemented fermented dairy products.
